# Characterization of constitutive and acid-induced outwardly rectifying chloride currents in immortalized mouse distal tubular cells

**DOI:** 10.1016/j.bbagen.2017.05.004

**Published:** 2017-08

**Authors:** William C. Valinsky, Rhian M. Touyz, Alvin Shrier

**Affiliations:** a Department of Physiology, McGill University, 3649 Promenade sir William Osler, Montreal, Quebec H3G 0B1, Canada; b Institute of Cardiovascular and Medical Sciences, University of Glasgow, BHF GCRC, 126 University Place, Glasgow G12 8TA, United Kingdom

**Keywords:** 2-APB, 2-aminoethoxydiphenyl borate, DCT, distal convoluted tubule, DIDS, 4,4′-diisothiocyanatostilbene-2,2′-disulfonate, FFA, flufenamic acid, HBE, human bronchial epithelial, HEK, human embryonic kidney, I-V, current-voltage, V_LJP_, liquid junction potential, MDCT, mouse distal convoluted tubule cell line, mIMCD-3, mouse inner medullary collecting duct cell line, NCC, Na^+^/Cl^−^ co-transporter, NPPB, 5-Nitro-2-(3-phenylpropylamino) benzoic acid, TRPM, transient receptor potential melastatin, VRAC, volume-regulated anion channel, Chloride current, Acid-induced chloride current, Acid-sensitive outwardly rectifying (ASOR) anion channel, Distal convoluted tubule, MDCT cells, mIMCD-3 cells

## Abstract

Thiazides block Na^+^ reabsorption while enhancing Ca^2+^ reabsorption in the kidney. As previously demonstrated in immortalized mouse distal convoluted tubule (MDCT) cells, chlorothiazide application induced a robust plasma membrane hyperpolarization, which increased Ca^2+^ uptake. This essential thiazide-induced hyperpolarization was prevented by the Cl^−^ channel inhibitor 5-Nitro-2-(3-phenylpropylamino) benzoic acid (NPPB), implicating NPPB-sensitive Cl^−^ channels, however the nature of these Cl^−^ channels has been rarely described in the literature. Here we show that MDCT cells express a dominant, outwardly rectifying Cl^−^ current at extracellular pH 7.4. This constitutive Cl^−^ current was more permeable to larger anions (Eisenman sequence I; I^−^ > Br^−^ ≥ Cl^−^) and was substantially inhibited by > 100 mM [Ca^2+^]_o_, which distinguished it from ClC-K2/barttin. Moreover, the constitutive Cl^−^ current was blocked by NPPB, along with other Cl^−^ channel inhibitors (4,4′-diisothiocyanatostilbene-2,2′-disulfonate, DIDS; flufenamic acid, FFA). Subjecting the MDCT cells to an acidic extracellular solution (pH < 5.5) induced a substantially larger outwardly rectifying NPPB-sensitive Cl^−^ current. This acid-induced Cl^−^ current was also anion permeable (I^−^ > Br^−^ > Cl^−^), but was distinguished from the constitutive Cl^−^ current by its rectification characteristics, ion sensitivities, and response to FFA. In addition, we have identified similar outwardly rectifying and acid-sensitive currents in immortalized cells from the inner medullary collecting duct (mIMCD-3 cells). Expression of an acid-induced Cl^−^ current would be particularly relevant in the acidic IMCD (pH < 5.5). To our knowledge, the properties of these Cl^−^ currents are unique and provide the mechanisms to account for the Cl^−^ efflux previously speculated to be present in MDCT cells.

## Highlights

• MDCT cells express a dominant NPPB-sensitive Cl^−^ current at pH 7.4.
• The constitutive Cl^−^ current (pH 7.4) does not arise from ClC-K2/barttin.
• MDCT cells also express an acid-induced NPPB-sensitive Cl^−^ current (pH < 5.5).
• Both the constitutive and acid-induced Cl^−^ currents are unique.
• mIMCD-3 cells express currents with similar biophysical properties.

## 1 Introduction

Hydrochlorothiazide is a diuretic that blocks the Na^+^/Cl^−^ co-transporter (NCC) in the distal convoluted tubule (DCT) [15]. Thiazides are also implicated in renal Ca^2+^ handling, as clinical reports have positively correlated thiazide treatment with hypocalciuria [69]. Moreover, in vivo microperfusion experiments have shown that thiazides increase Ca^2+^ reabsorption in rat DCT cells [11], and investigations of immortalized mouse DCT (MDCT) cells have suggested that thiazides increase Ca^2+^ transport by hyperpolarizing the plasma membrane [21]. While the thiazide effect on Ca^2+^ is now considered to result from changes in passive reabsorption in the proximal tubule [52], it is notable that the DCT based mechanism is dependent on membrane hyperpolarization [21], and would therefore increase the transport of any ion with a positive reversal potential.

A crucial detail of the thiazide-stimulated hyperpolarization was its cessation by the Cl^−^ channel inhibitor 5-Nitro-2-(3-phenylpropylamino) benzoic acid (NPPB). It was suggested that thiazides blocked the NCC, eliminating Cl^−^ entry. However, intracellular Cl^−^ continued to exit MDCT cells through NPPB-sensitive Cl^−^ channels, decreasing [Cl^−^]_i_. This reduced the impact of Cl^−^ on the membrane reversal potential and shifted MDCT cells towards the more negative K^+^ reversal potential [21]. The proposed link between Cl^−^ efflux channels and the NCC is particularly intriguing since intracellular Cl^−^ depletion is a known activator of the NCC [55]. Thus, a relationship may exist between Cl^−^ efflux, NCC function, and membrane voltage. Since MDCT cells express the NCC and associated interacting proteins [2], [20], [23], [24], [37], the NPPB-sensitive Cl^−^ channels expressed in MDCT cells may be physiologically relevant.

To date, the only electrophysiological data available for MDCT cells shows an outwardly rectifying current that is inhibited by 500 μM 2-aminoethoxydiphenyl borate (2-APB) [42], a non-selective cation channel blocker [10], [39], [71], [77]. While this current was attributed to transient receptor potential melastatin 7 (TRPM7), the shape of the current-voltage (I-V) relationship is similar to that of ClC-K2(b)/barttin [16], a Cl^−^ channel expressed on DCT basolateral membranes [7], [29], [38]. In addition, the nephron experiences a varying degree of pH in the filtrate. Thus, acid-induced currents would be relevant in this tissue.

Acid-induced outwardly rectifying Cl^−^ currents have been found in neurons, cardiac myocytes, blood cells, and epithelial cells [1], [8], [19], [40], [41], [47], [48], [53], [64], [65], [75], [78]. These studies primarily examined biophysical properties, and presently little is known about the biological role or molecular entity of these currents. The volume-regulated anion channel (VRAC) was the first proposed molecular candidate [53], however further biophysical analysis showed that VRAC and acid-induced Cl^−^ currents comprise different conductances [41]. More recent evidence further separated these currents, as a molecular component of VRAC, LRRC8A or SWELL1 [56], [74], has no apparent role in acid-induced Cl^−^ currents [62], [63]. Furthermore, multiple members of the LRRC8 family [62], [63], ClC family [1], [8], [54], and TMEM16 family [8] have been ruled out as possible molecular components of the acid-induced Cl^−^ current.

In this study, we characterized the currents present in MDCT cells at both neutral and acidic extracellular pH. We show that MDCT cells express an NPPB-sensitive outwardly rectifying Cl^−^ current at pH 7.4 and an even larger NPPB-sensitive outwardly rectifying Cl^−^ current at pH < 5.5. We further show that immortalized cells from the terminally located and most highly acidic (pH < 5.5) inner medullary collecting duct (mIMCD-3) also express similar outwardly rectifying and acid-induced currents. Our analysis of these Cl^−^ currents suggests they are unique and would account for the Cl^−^ efflux previously implicated in the hyperpolarizing response of thiazides on MDCT cells.

## 2 Material and methods

### 2.1 Cell culture

The previously established MDCT cell line was provided by Dr. David Clapham, Harvard University, Cambridge, MA and by Dr. Lixia Yue, Department of Cell Biology, University of Connecticut, Farmington, CT. The previously established mIMCD-3 cell-line was provided by Dr. Reza Sharif-Naeini, McGill University, QC. Cells were grown in Dulbecco's Modified Eagle Medium (low glucose; ThermoFisher Scientific Gibco, Waltham, MA) supplemented with 10% FBS (Wisent Bioproducts, St-Bruno, QC), and 100 U/mL penicillin and 100 μg/mL streptomycin (ThermoFisher Scientific Gibco). Cells were cultured at 37 °C in 5% CO_2_. Cell media was changed every 3–4 days and cells were passaged every 4–5 days via trypsinization.

### 2.2 RNA isolation, cDNA synthesis, and real-time quantitative PCR (RT-qPCR)

RNA was extracted using a NucleoSpin RNA II kit (Macherey-Nagel, Bethlehem, PA) and cDNA was synthesized using an iScript kit (Bio-Rad, Hercules, CA). RT-qPCR was performed using a SsoFast Evagreen Supermix with low ROX kit (Bio-Rad) and read-out on a LightCycler 96 (Roche, Penzberg, Germany) or an Illumina Eco (Illumina, San Diego, CA). The program used consisted of a pre-incubation (95 °C, 120 s) followed by 45 cycles of 2-step amplification (95 °C for 10 s, 60 °C for 30 s). After completion of the amplification, a melt curve was generated by increasing temperature from 65 °C to 95 °C at a rate of 0.2 °C/s. The derivative of the melt curve was used to assess product purity. Expression was normalized to β-actin. The primers used were the following:

mouse TRPM7 5′-TTCACTCGGTGCAAGAAAGCTG-3′ (forward).
mouse TRPM7 5′-GGTCTATCTCGTAACCAATCCGGT-3′ (reverse).
mouse TRPM6 5′-TCCGTCCATGGGGGTCTTCA-3′ (forward).
mouse TRPM6 5′-CCCCAACGTGCTTGGACACT-3′ (reverse).
mouse β-actin 5′-CCTTCCTTCTTGGGTATGGA-3′ (forward).
mouse β-actin 5′-TGCTAGGAGCCAGAGCAGTA-3′ (reverse).

### 2.3 RNA interference

TRPM7 knockdown was performed using SMARTpool small interfering RNA (siRNA) to murine TRPM7 (ThermoFisher Scientific Dharmacon). MDCT cells were plated in a 35 mm plastic cell culture dish, transfected with 100 nM siRNA using oligofectamine (ThermoFisher Scientific Invitrogen) for 6 h in OPTIMEM (ThermoFisher Scientific Gibco), and utilized 24 h after transfection. Prolonged periods of TRPM7 siRNA transfection reduced cellular viability, and therefore a 24 h knockdown period was used. TRPM7 knockdown was verified using RT-qPCR. For electrophysiological experiments, cells were co-transfected with siGLO green transfection indicator (Thermo Scientific Dharmacon), which was used to select transfected cells.

### 2.4 Electrophysiology

Cells were plated on Poly-l-Lysine coated 8 mm coverslips, placed in the perfusion chamber of an inverted microscope (Zeiss Axiovert S100TV), and perfused at a rate of 1–2 mL/min with the solutions presented in Table 1. Borosilicate patch pipettes (AM-Systems, Carlsborg, WA) were prepared using a microprocessor-controlled, multistage puller (P97; Sutter Instruments, Navoto, CA), and fire-polished to a resistance of ~ 2–4 MΩ. All experiments were performed at room temperature (~ 21 °C).

**Table 1:** Patch solutions and liquid junction potential (V_LJP_).

#	Extracellular (outside) solution (o) (mM)	Intracellular solution (i) (mM)	V_LJP_ (mV)
I	145 NaCl, 5.4 KCl, 1.8 CaCl_2_, 1.0 MgCl_2_, 5.0 HEPES. pH 7.4, 6.0, 5.0, or 4.0. Referred to as “Tyrodes”.	130 CsCl, 10 Cs_4_BAPTA, 10 NaCl, 10 HEPES. pH 7.2.	+2
II	145 NaCl, 5.4 KCl, 2.8 CaCl_2_, 5.0 HEPES. pH 7.4 or 5.0.	130 CsCl, 10 Cs_4_BAPTA, 10 NaCl, 10 HEPES. pH 7.2.	+2
III	150 NMDG^+^, 2.8 CaCl_2_, 5.0 HEPES. pH 7.4 or 5.0.	130 CsCl, 10 Cs_4_BAPTA, 10 NaCl, 10 HEPES. pH 7.2.	+8
IV	110 CaCl_2_, 5.0 HEPES. pH 7.4 or 5.0.	130 CsCl, 10 Cs_4_BAPTA, 10 NaCl, 10 HEPES. pH 7.2.	+5
V	145 NaCl, 5.4 KCl, 2.8 MgCl_2_, 5.0 HEPES. pH 7.4 or 5.0.	130 CsCl, 10 Cs_4_BAPTA, 10 NaCl, 10 HEPES. pH 7.2.	+2
VI	150 NMDG^+^, 2.8 MgCl_2_, 5.0 HEPES. pH 7.4 or 5.0.	130 CsCl, 10 Cs_4_BAPTA, 10 NaCl, 10 HEPES. pH 7.2.	+8
VII	110 MgCl_2_, 5.0 HEPES. pH 7.4 or 5.0.	130 CsCl, 10 Cs_4_BAPTA, 10 NaCl, 10 HEPES. pH 7.2.	+7
VIII	75 NaCl, 70 NMDG^+^, 1.8 CaCl_2_, 1.0 MgCl_2_, 5.0 HEPES. pH 7.4 or 5.0.	130 CsCl, 10 Cs_4_BAPTA, 10 NaCl, 10 HEPES. pH 7.2.	+4
IX	25 NaCl, 120 NMDG^+^, 1.8 CaCl_2_, 1.0 MgCl_2_, 5.0 HEPES. pH 7.4 or 5.0.	130 CsCl, 10 Cs_4_BAPTA, 10 NaCl, 10 HEPES. pH 7.2.	+7
X	145 Na^+^ Glutamate, 5.4 KCl, 1.8 CaCl_2_, 1.0 MgCl_2_, 5.0 HEPES. pH 7.4 or 5.0.	130 CsCl, 10 Cs_4_BAPTA, 10 NaCl, 10 HEPES. pH 7.2.	−2
XI	145 NaCl, 5.4 KCl, 1.8 CaCl_2_, 1.0 MgCl_2_, 5.0 HEPES. pH 7.4 or 5.0.	130 K^+^ methanesulfonate (CH_3_SO_3_^−^), 10 Cs_4_BAPTA, 10 NaCl, 10 HEPES. pH 7.2.	+10
XII	145 NaBr, 5.4 KCl, 1.8 CaCl_2_, 1.0 MgCl_2_, 5.0 HEPES. pH 7.4 or 5.0.	130 CsCl, 10 Cs_4_BAPTA, 10 NaCl, 10 HEPES. pH 7.2.	+2
XIII	145 NaI, 5.4 KCl, 1.8 CaCl_2_, 1.0 MgCl_2_, 5.0 HEPES. pH 7.4 or 5.0.	130 CsCl, 10 Cs_4_BAPTA, 10 NaCl, 10 HEPES. pH 7.2.	+2
XIV	155 NaCl, 5.4 KCl, 5.0 HEPES. pH 7.4.	130 CsCl, 10 Cs_4_BAPTA, 10 NaCl, 10 HEPES. pH 7.2.	+2

Composition of extracellular solution (2nd column), intracellular solution (3rd column), and determined V_LJP_ (4th column). Solution combinations in the text are presented as Roman numerals (1st column).

Whole-cell currents were recorded using an Axopatch 200B amplifier (Axon Instruments, Sunnyvale, CA) coupled to a CV 203BU headstage (Axon Instruments). Command pulses were generated by a Digidata 1440A (Axon Instruments) via pClamp 10.4 software. Data were acquired at 20 kHz and low pass filtered at 2 kHz. Prior to the formation of a GΩ seal, currents were corrected for pipette (fast) capacitance. Upon formation of the whole cell-configuration, cell capacitance (pF) was determined using a 30 ms, 10 mV depolarizing pulse from a holding potential of −80 mV, at 2 Hz. Currents were corrected for whole-cell capacitance and series resistance compensated to 80%. All recorded cells had access resistances below 10 MΩ.

**Fig. 1:**
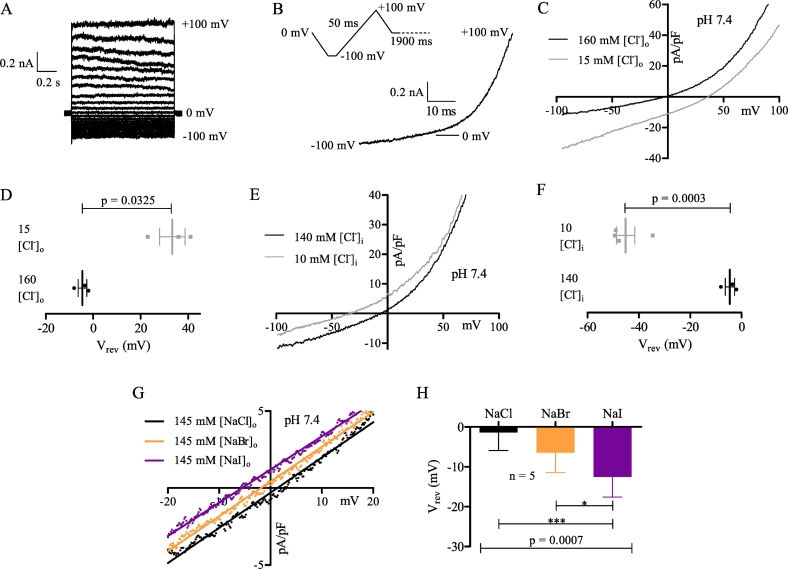
MDCT cells express a dominant outwardly rectifying Cl^-^ current at neutral pH. (A) Whole-cell recording of an MDCT cell elicited from a holding potential of 0 mV and stepped in +10 mV increments from -100 mV to +100 mV in extracellular solution I (oI – see Table 1). (B) Whole-cell recording of an MDCT cell elicited from the 50 ms ramp protocol (-100 mV to +100 mV; oI) shown in the inset. (C) Representative MDCT current recorded with 160 mM [Cl^-^]_o_ (black; oI) or 15 mM [Cl^-^]_o_ (grey; oX). (D) Mean reversal potential of MDCT currents using the solutions in C (n = 3). (E) Representative MDCT current recorded with 140 mM [Cl^-^]_i_ (black; iI) or 10 mM [Cl^-^]_i_ (grey; iXI). (F) Mean reversal potential of MDCT currents with 140 mM (n = 3) or 10 mM (n = 4) [Cl^-^]_i_. (G) Representative MDCT current recorded with 145 mM [NaCl]_o_ (black; oI), 145 mM [NaBr]_o_ (orange; oXII), or 145 mM [NaI]_o_ (violet; oXIII). Data were fit by linear regression. (H) Mean reversal potential (best-fit y intercepts) of MDCT currents using the solutions in G (n = 5). Whole-cell current traces are presented in nA and representative samples of current density in pA/pF. Mean reversal potentials (mean ± SEM) were compared via a paired two-tailed Student's *t*-test (D), an unpaired two-tailed Student's *t*-test (F), or a repeated measures one-way ANOVA with post-Bonferroni tests (H). Data were considered significant when p < 0.05. * refers to p = 0.05 and *** to p = 0.001.

Recordings of MDCT cells using a step protocol from −100 mV to +100 mV in +10 mV increments at 1 Hz per step (Fig. 1A) showed that P/N leak subtraction protocols could not be utilized since currents were active at all voltages tested (except for reversal). Additionally, currents rapidly activated and did not inactivate during test pulses (Fig. 1A), enabling the use of a 50 ms ramp from −100 mV to +100 mV at 0.5 Hz (Fig. 1B), where data could be collected every 0.2 mV. Unless stated otherwise, all reported currents were recorded using the ramp protocol.

Table 1 lists the composition and combination of solutions used along with the measured liquid junction potential (V_LJP_; mV) [51]. The V_LJP_ was corrected offline using the formula: V_membrane_ = V_pipette_ − V_LJP_. In most conditions, the dominant intracellular cation was Cs^+^, consistent with the prior electrophysiological characterization of MDCT cells [42]. Solution pH was adjusted with HCl or the hydroxide salt of the cation with the largest concentration. Osmolarity was routinely 285 mOsm, as determined by a vapor pressure osmometer (Wescor, Utah). Where appropriate, an electronic valve controller (VC-8, Warner Instruments, Hamden CT) was used to switch solutions.

### 2.5 Pharmacology

A variety of channel blocking drugs were assessed including: 2-aminoethoxydiphenyl borate (2-APB; 200 μM) disodium 4,4′-diisothiocyanatostilbene-2,2′-disulfonate (DIDS; 100 μM), 5-Nitro-2-(3-phenylpropylamino) benzoic acid (NPPB; 100 µM), flufenamic acid (FFA; 100 μM), or furosemide (100 μM, 1 mM). All compounds were purchased from Sigma Aldrich, and dissolved in solvents recommended by Sigma Aldrich.

### 2.6 Data analysis

All experimental data were generated from a minimum of 3 experiments. For RT-qPCR data, the label “n” refers to the number of experiments, each performed in triplicate. The Cq for each triplicate was averaged. Averaged Cqs for genes of interest were subtracted by the average Cq for β-actin, generating ΔCqs. Relative expression was determined using the 2^−ΔΔCq^ methodology [45].

For electrophysiological data, the label “n” refers to the number of cells recorded, each from a separate dish. In I-V plots, cellular ionic currents (pA) were normalized for cell size (pF), expressed as pA/pF (current density), and presented in 0.2 mV increments. Analyzed currents were selected after a stable peak current was identified at +100 mV and −100 mV in pClamp.

To estimate the relative permeability of anions to Cl^−^ (P_x_/P_Cl_), the following formula was used [6], [59]:

$$E_{\mathrm{rev}}=-58\log\left(\frac{{\left[Cl^{-}\right]}_{o}\times P_{\mathrm{Cl}}+{\left[X^{-}\right]}_{o}\times P_{x}}{{\left[Cl^{-}\right]}_{i}\times P_{\mathrm{Cl}}+{\left[X^{-}\right]}_{i}\times P_{x}}\right)$$

For extracellular Cl^−^ substitution, where [X]_*i*_ = 0 and P_Cl_ = 1:

$$P_{x}=\frac{10^{\frac{E_{\mathrm{rev}}}{-58}}\times {\left[Cl^{-}\right]}_{i}-{\left[Cl^{-}\right]}_{o}}{{\left[X^{-}\right]}_{o}}$$

For intracellular Cl^−^ substitution, where [X]_*o*_ = 0 and P_Cl_ = 1:

$$P_{x}=\frac{{{\left[Cl^{-}\right]}_{o}-10}^{\frac{E_{\mathrm{rev}}}{-58}}\times {\left[Cl^{-}\right]}_{i}}{{10^{\frac{E_{\mathrm{rev}}}{-58}}\times \left[X^{-}\right]}_{i}}$$

### 2.7 Statistical analysis

Grouped data are presented as means or mean ± standard error mean (SEM). Statistical analyses were performed using GraphPad Prism 5.0. For reversal potential identification of pH 7.4 currents, linear regression analysis was performed using y = mx + b (intercept), and extrapolated data were best-fit. Statistical comparisons were made using one-way ANOVA with post-Bonferroni tests, repeated measures one-way ANOVA with post-Bonferroni tests, paired two-tailed Student's *t*-tests, unpaired two-tailed Student's *t*-tests, or Mann-Whitney *U* tests. The statistical methodology chosen is indicated in each figure legend. Statistical significance was determined by p < 0.05. Precise p values are depicted in most plots. Occasionally, asterisks are used to indicate statistical significance; * refers to p ≤ 0.05, ** refers to p ≤ 0.01, *** refers to p ≤ 0.001, **** refers to p ≤ 0.0001.

## 3 Results

### 3.1 A prominent Cl^−^ current is endogenously expressed in MDCT cells at neutral pH

Using a voltage clamp step protocol, we found that MDCT macroscopic currents were constitutively active, time-independent, and outwardly rectifying (Fig. 1*A*). Since the current was time-independent, we used a ramp protocol repeated every 2 s to monitor changes in the I-V relationship (Fig. 1*B*). To assess the ion permeation characteristics of macroscopic currents, ion substitution experiments were performed and changes in the reversal potential were examined. We determined that MDCT cells exhibit a high permeability to Cl^−^. When [Cl^−^]_o_ was reduced from 160 mM to 15 mM by substitution with glutamate^−^ (oI to oX – see Table 1), a +37.9 ± 7.0 mV shift in the reversal potential was observed (Fig. 1C, D). Conversely, when [Cl^−^]_i_ was reduced from 140 mM to 10 mM by replacement with methanesulfonate (CH_3_SO_3_^−^), a highly significant −40.6 ± 4.5 mV shift in the reversal potential was observed (iI to iXI) (Fig. 1E, F). Furthermore, the reversal potential in normal Tyrodes at pH 7.4 (oI; including data presented in Fig. 1G, H) was −2.6 ± 2.9 mV (n = 8). This is extremely close to the Nernst predicted reversal of −3.4 mV for a pure Cl^−^ current, indicating that the pH 7.4 current in MDCT cells is mostly Cl^−^ based.

It was notable that reversal potentials after the glutamate^−^ and CH_3_SO_3_^−^ substitutions were less than the predicted Nernst values (+59.5 mV and −73.3 mV, respectively). However, many Cl^−^ currents are not only anion permeable [1], [6], [8], [16], [17], [19], [41], [46], [53], [57], [59], [78], but are permeable to negatively charged molecules such as glutamate^−^ [6], [59] and CH_3_SO_3_^−^ [28], [31], [68].

We therefore evaluated whether the constitutive Cl^−^ current was multi-anion permeable and delineated the selectivity sequence using a bi-ionic substitution protocol whereby 145 mM [NaCl]_o_ was replaced with 145 mM [NaBr]_o_, which was then replaced with 145 mM [NaI]_o_ (oI to oXII to oXIII). In the sample trace provided (Fig. 1*G*), best-fit y intercepts were +1.3 mV for NaCl, −1.9 mV for NaBr, and −5.6 mV NaI over the linear range of the I-V relationship between −20 mV and +20 mV. These best-fit trends held for all cells (Fig. 1H), and thus the constitutive Cl^−^ current is multi-anion permeable with a selectivity sequence of I^−^ > Br^−^ ≥ Cl^−^. We further calculated the permeability of all anions and molecules tested relative to Cl^−^ (Table 2), and found that the selectivity sequence was I^−^ > Br^−^ ≥ Cl^−^>>>>glutamate^−^ ≥ CH_3_SO_3_^−^. For the anions, this selectivity sequence corresponds to Eisenman sequence I [13], [14], indicating that ion permeation requires ion dehydration.

**Table 2:** Effect of anion substitution on reversal potential (mV) and relative permeability.

	pH 7.4 (constitutive)			pH 5.0 (acid-induced)		
	n	ΔE_rev_ (mV)	P_x_/P_Cl_	n	ΔE_rev_ (mV)	P_x_/P_Cl_
I^−^	5	−11.1 ± 2.3	1.64 ± 0.16	5	−13.0 ± 1.5	1.76 ± 0.11
Br^−^	5	−5.1 ± 1.2	1.25 ± 0.07	5	−5.2 ± 0.7	1.26 ± 0.04
Glutamate^−^	3	+37.9 ± 7.0	0.16 ± 0.07	5	+26.2 ± 2.1	0.29 ± 0.03
CH_3_SO_3_^−^	3+	−40.6 ± 4.5	0.14	5	−28.9 ± 1.4	0.34

For extracellular substitutions, Cl^−^ was replaced with I^−^, Br^−^, or glutamate^−^. E_Rev_ and P_x_/P_Cl_ were calculated from 5 paired recordings. For intracellular substitutions, Cl^−^ (n = 3) and CH_3_SO_3_^−^ (n = 4) reversal potentials were determined from different cells. E_Rev_ and P_x_/P_Cl_ were thus unpaired observations and calculated from the mean data of each group.

### 3.2 Effects of cations on the constitutive Cl^−^ current at neutral pH

**Fig. 2:**
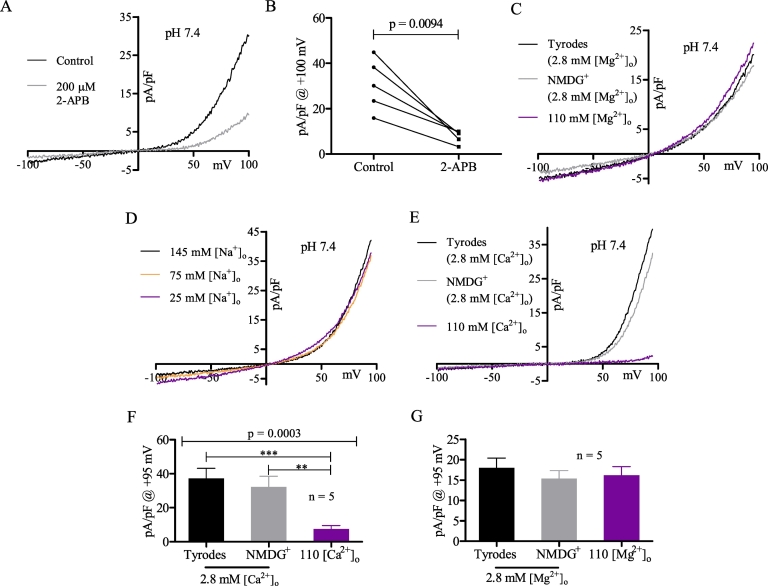
The constitutive Cl^-^ current is 2-APB and [Ca^2+^]_o_ inhibited. (A) Representative MDCT current before (black) and during (grey) maximal 200 µM 2-APB inhibition (oI). (B) Plot of current density at +100 mV using the conditions described in A (n = 5). (C) Representative MDCT current recorded with extracellular solutions containing (mM): 2.8 Mg^2+^/145 Na^+^/5.4 K^+^ (black; oV), 2.8 Mg^2+^/150 NMDG^+^ (grey; oVI), or 110 Mg^2+^ (violet; oVII). (D) Representative MDCT current recorded in extracellular solutions where 145 mM [Na^+^]_o_ (black; oI) was reduced to 75 mM [Na^+^]_o_ (orange; oVIII), and subsequently to 25 mM [Na^+^]_o_ (violet; oIX) by replacement of Na^+^ with equimolar NMDG^+^. All other salts were unchanged. (E) Representative MDCT current recorded with extracellular solutions containing (mM): 2.8 Ca^2+^/145 Na^+^/5.4 K^+^ (black; oII), 2.8 Ca^2+^/150 mM NMDG^+^ (grey; oIII), or 110 Ca^2+^ (violet; oIV). (F) Plot of current density at +95 mV using the solutions described in E (n = 5). (G) Plot of current density at +95 mV using the solutions described in C (n = 5). Currents were statistically compared using a paired two-tailed Student's *t*-test (B) or a repeated measures one-way ANOVA with post-Bonferroni tests (F, G) (mean ± SEM). Data were considered significant when p < 0.05. ** refers to p = 0.01 and *** to p = 0.001.

Previously it was proposed that the 2-APB inhibited outwardly rectifying current of MDCT cells was carried by TRPM7 [42]. We confirmed this inhibitory effect of 2-APB on MDCT currents (Fig. 2A, B) and also confirmed that TRPM7 was expressed using RT-qPCR (Fig. 8A, B). To assess whether TRPM7 contributed to MDCT currents, Mg^2+^ supplementation experiments were conducted since TRPM7 currents are considerably inhibited by ≥ 100 mM [Mg^2+^]_o_ [42], [49], [50]. When [Mg^2+^]_o_ was increased from 2.8 mM to 110 mM (oV to oVI to oVII), we observed no changes in current magnitude or reversal potential (Fig. 2C, G), which strongly argues against the presence of a major TRPM7 current.

To assess potential contributions from other cationic currents, we examined the effect of increasing [Ca^2+^]_o_ from 2.8 mM to 110 mM (oII to oIII to oIV), or decreasing [Na^+^]_o_ from 145 mM to 25 mM (oI to oVIII to oIX). These conditions did not affect the reversal potential of MDCT cells (Fig. 2D, E), indicating that MDCT cells express a dominant constitutive Cl^−^ current. However, peak Cl^−^ current magnitude (+95 mV) was substantially reduced by 110 mM [Ca^2+^]_o_ (Fig. 2F). It was notable that 110 mM [Mg^2+^]_o_ was unable to reproduce this effect (Fig. 2G), indicating that the Ca^2+^ mediated inhibition cannot be attributed to charge shielding [27].

### 3.3 The constitutive Cl^−^ current is inhibited by Cl^−^ channel blockers, including NPPB

**Fig. 3:**
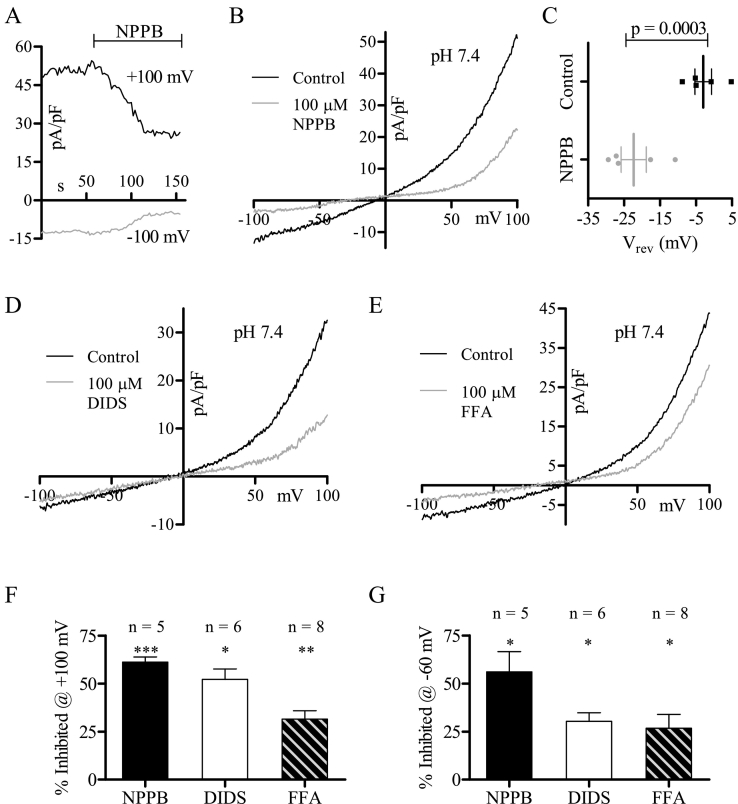
The constitutive Cl^-^ current is inhibited by NPPB, DIDS, and FFA. (A) Representative trace of MDCT currents recorded over time during 100 µM NPPB superfusion at +100 mV and -100 mV (oI). (B) Representative MDCT current before (black) and during (grey) maximal 100 µM NPPB blockade (oI). (C) Mean reversal potential of MDCT currents in conditions B (n = 5). (D) Representative MDCT current before (black) and during (grey) maximal 100 µM DIDS blockade (oI). (E) Representative MDCT current before (black) and during (grey) maximal 100 µM FFA blockade (oI). (F) Mean percentage of MDCT current inhibited at +100 mV by NPPB (n = 5), DIDS (n = 6), and FFA (n = 8). (G) Mean percentage of MDCT current inhibited at -60 mV by NPPB (n = 5), DIDS (n = 6), and FFA (n = 8). Data were statistically compared via paired two-tailed Student's *t*-tests (mean ± SEM) and were considered significant when p < 0.05. * refers to p = 0.05, ** to p = 0.01, *** to p = 0.001.

The Cl^−^ efflux required for the thiazide-stimulated membrane hyperpolarization in MDCT cells was NPPB-sensitive [21]. Thus, we investigated the sensitivity of the constitutive Cl^−^ current to NPPB along with other Cl^−^ channel inhibitors (DIDS, FFA) [43], [44]. NPPB (100 μM) blocked the constitutive Cl^−^ current (Fig. 3A, B, F, G) and shifted the reversal potential (Fig. 3C), indicative of a compound that effectively eliminates the Cl^−^ current. Importantly, this block was prominent over the range of reported MDCT resting potentials (−30 mV to −75 mV) [12], [22], [25], [26], [35] (Fig. 3B, G), which is directly relevant for the thiazide-stimulated hyperpolarization of MDCT cells. DIDS (100 μM; Fig. 3D, F, G) and FFA (100 μM; Fig. 3E–G) also blocked the constitutive Cl^−^ current, however the FFA blockade was the lowest in magnitude (Fig. 3F, G).

### 3.4 Extracellular acidification induces a large outwardly rectifying Cl^−^ current

**Fig. 4:**
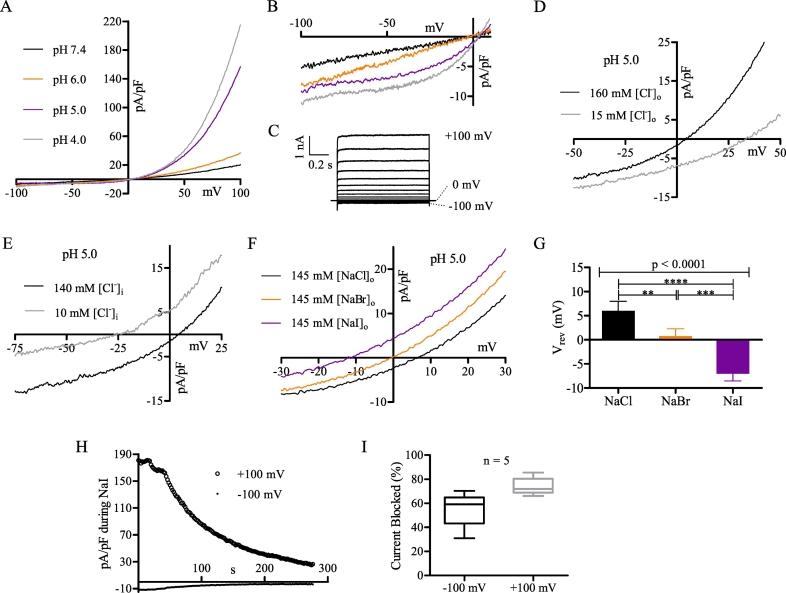
Extracellular acidification induces an outwardly rectifying Cl^-^ current. (A) Representative MDCT current recorded in extracellular solution (oI) at pH 7.4 (black), pH 6.0 (orange), pH 5.0 (violet), or pH 4.0 (grey). (B) Expanded I-V relationship of A from -100 mV to +5 mV. (C) Whole-cell recording elicited from a holding potential of 0 mV and stepped in +10 mV increments from -100 mV to +100 mV at pH 5.0. (D) Representative MDCT current recorded with 160 mM [Cl^-^]_o_ (black; oI) or 15 mM [Cl^-^]_o_ (grey; oX). (E) Representative MDCT current recorded with 140 mM [Cl^-^]_i_ (black; iI) or 10 mM [Cl^-^]_i_ (grey; iXI). (F) Representative MDCT current recorded with 145 mM [NaCl]_o_ (black; oI), 145 mM [NaBr]_o_ (orange; oXII), or 145 mM [NaI]_o_ (violet; oXIII). (G) Mean reversal potential of MDCT currents using the solutions in F (n = 5). (H) Representative trace of MDCT current at -100 mV and +100 mV during superfusion of 145 mM [NaI]_o_ (oXIII). (I) Mean inhibition (peak; n = 5) during 145 mM [NaI]_o_ superfusion at +100 mV (grey) and -100 mV (black). Whole-cell traces are presented in nA and representative samples of current density in pA/pF. Reversal potentials (mean ± SEM) were statistically compared using a repeated measures one-way ANOVA with post-Bonferroni tests. Data were considered significant when p < 0.05. ** refers to p = 0.01, *** to p = 0.001, **** to p = 0.0001.

Since the mammalian renal system is highly dynamic and subject to varying pH in the urinary filtrate, we studied MDCT cells over a broad range of extracellular pH, extending from pH 4.0 to pH 7.4 (oI). We observed that extracellular acidification to pH 5.0 induced a large, outwardly rectifying current at positive potentials (Fig. 4A). At negative potentials, the I-V relationship shifted from linear at pH 7.4 and 6.0 to outwardly rectifying at pH 5.0 and pH 4.0 (Fig. 4B). When examined using a step protocol, the acid-induced current (pH 5.0) was time-independent and non-inactivating (Fig. 4C). Using the Cl^−^ substitution protocols described above (3.1; Fig. 1C–F), we found that the acid-induced current is Cl^−^ permeable. Reduction of [Cl^−^]_o_ from 160 mM to 15 mM (oI to oX) at pH 5.0 resulted in a significant +26.3 ± 2.1 mV shift of the reversal potential (Fig. 4D), whereas reduction of [Cl^−^]_i_ from 140 mM to 10 mM (iI to iXI) caused a significant −28.9 ± 1.4 mV shift in the reversal potential (Fig. 4E). In performing identical bi-ionic substitutions experiments to those described above (3.1; Fig. 1G, H), we determined that the acid-induced current was also anion permeable with a selectivity sequence of I^−^ > Br^−^ > Cl^−^ (Fig. 4F, G). Based upon the calculated relative permeability data (Table 2), the selectivity sequence is I^−^ > Br^−^ > Cl^−^ >>> CH_3_SO_3_^−^ ≥ glutamate^−^, which slightly differs from the constitutive Cl^−^ current. We also noted that prolonged perfusion of 145 mM [NaI]_o_ substantially reduced the acid-induced Cl^−^ current (74 ± 3.2% at +100 mV, 55 ± 6.5% at −100 mV; Fig. 4H, I), an effect that was not observed for the constitutive Cl^−^ current.

### 3.5 Effects of cations on the acid-induced Cl^−^ current

**Fig. 5:**
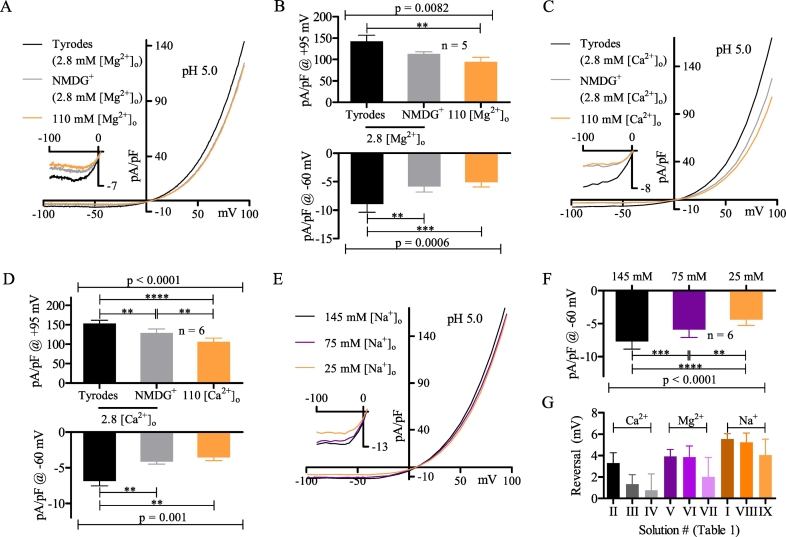
MDCT acid-induced currents are monovalent cation sensitive. (A) Representative MDCT current recorded with extracellular solutions containing (mM): 2.8 Mg^2+^/145 Na^+^/5.4 K^+^ (black; oV), 2.8 Mg^2+^/150 NMDG^+^ (grey; oVI), or 110 Mg^2+^ (orange; oVII). Inward current and reversal potential are shown in the inset. (B) Mean current density at +95 mV (top) and -60 mV (bottom) using the solutions in A (n = 5). (C) Representative MDCT current recorded with extracellular solutions containing (mM): 2.8 Ca^2+^/145 Na^+^/5.4 K^+^ (black; oII), 2.8 Ca^2+^/150 mM NMDG^+^ (grey; oIII), or 110 Ca^2+^ (orange; oIV). Inward current and reversal potential are shown in the inset. (D) Mean current density at +95 mV (top) and -60 mV (bottom) using the solutions in C (n = 6). (E) Representative MDCT current recorded in extracellular solutions where 145 mM [Na^+^]_o_ (black; oI) was reduced to 75 mM [Na^+^]_o_ (violet; oVIII), and subsequently to 25 mM [Na^+^]_o_ (orange; oIX) by replacement of Na^+^ with equimolar NMDG^+^. Inward current and reversal potential are shown in the inset. (F) Mean current density at -60 mV using the solutions in E (n = 6). (G) Mean reversal potential for the Ca^2+^ (solutions C; n = 6), Mg^2+^ (solutions A; n = 5), and Na^+^ (solutions E; n = 6) extracellular substitution experiments. Each ion triplet comprises a paired data set. Data were statistically compared via a repeated measures one-way ANOVA with post-Bonferroni tests (mean ± SEM) and were considered significant when p < 0.05. ** refers to p = 0.01, *** to p = 0.001, **** to p = 0.0001.

The mean reversal potential in normal Tyrodes at pH 5.0 (oI) was +3.9 ± 1.5 mV (n = 10), which is positive to both the predicted Nernst value for a pure Cl^−^ current (−3.4 mV) and the data obtained at pH 7.4 (−2.6 ± 2.9 mV). One possible explanation is that increased [H^+^]_o_ activated cation currents. To test this possibility, we performed cation substitution experiments using conditions described above (3.2; Fig. 2). At pH 5.0, shifts in [Mg^2+^]_o_ (oV to oVI to oVII), [Ca^2+^]_o_ (oII to oIII to oIV), or [Na^+^]_o_ (oI to oVIII to oIX) did not significantly affect reversal potentials (Fig. 5G), suggesting that H^+^-activated cation currents could only represent a small fraction of the total macroscopic current. In addition, replacement of extracellular monovalent cations with NMDG^+^ caused a modest but statistically significant decrease of current magnitude at +95 mV and −60 mV (Fig. 5A–D). Sequential reduction of [Na^+^]_o_ from 145 mM to 75 mM to 25 mM (oI to oVIII to oIX) also caused a modest but statistically significant reduction of current at −60 mV (Fig. 5E, F). We noted that the effective elimination of the constitutive Cl^−^ current by > 100 mM [Ca^2+^]_o_ (Fig. 2E,F) was not observed for the acid-induced Cl^−^ current (Fig. 5C, D).

### 3.6 Pharmacology of the MDCT acid-induced Cl^−^ current

**Fig. 6:**
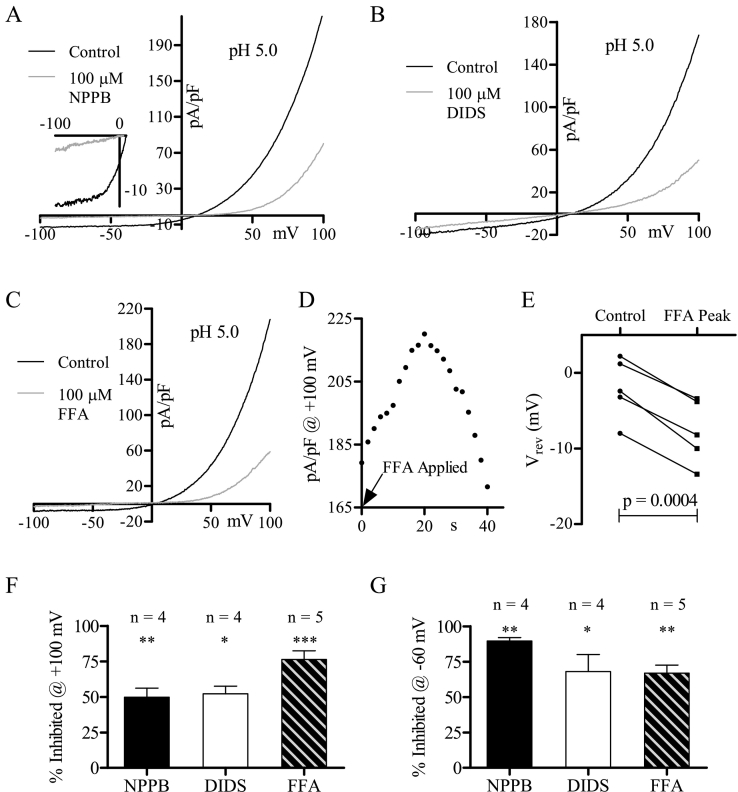
The acid-induced Cl^-^ current is inhibited by DIDS, NPPB, and FFA. (A) Representative MDCT current before (black) and during (grey) maximal 100 µM NPPB blockade (oI). The inset shows current from -100 mV to ~ 0 mV. (B) Representative MDCT current before (black) and during (grey) maximal 100 µM DIDS blockade (oI). (C) Representative MDCT current before (black) and during (grey) maximal 100 µM FFA blockade (oI). (D) Representative trace of MDCT current recorded over time during FFA superfusion (+100 mV). (E) Plot of reversal potential before and during maximal current enhancement from FFA superfusion. (F) Mean percentage of MDCT current inhibited at +100 mV by NPPB (n = 4), DIDS (n = 4), and FFA (n = 5). (G) Mean percentage of MDCT current inhibited at -60 mV by NPPB (n = 4), DIDS (n = 4), and FFA (n = 5). Data were statistically compared via paired two-tailed Student's *t*-tests (mean ± SEM) and were considered significant when p < 0.05. * refers to p = 0.05, ** to p = 0.01, *** to p = 0.001.

While it is possible that the acid-induced Cl^−^ current resulted from enhanced activation of the constitutive Cl^−^ current, it exhibited striking similarity to a group of previously discovered acid-induced Cl^−^ currents [1], [8], [19], [40], [41], [47], [48], [53], [64], [65], [75], [78]. To further compare the MDCT currents, we studied the effects of NPPB (100 μM), DIDS (100 μM), and FFA (100 μM) on the acid-induced Cl^−^ current (Fig. 6). It was notable that NPPB blocked nearly all of the acid-induced current at negative voltages (Fig. 6A, G), indicating that NPPB would significantly reduce the acid-induced Cl^−^ current at physiological potentials. Differences between the constitutive and acid-induced Cl^−^ currents became apparent with FFA. The acid-induced Cl^−^ current was far more sensitive to FFA, as current recorded at +100 mV was reduced by 76.3 ± 6.1% (Fig. 6F) compared to 31.5 ± 4.3% for the constitutive Cl^−^ current (Fig. 3F). Furthermore, prior to inhibition, FFA actually enhanced the acid-induced Cl^−^ current (Fig. 6D) and shifted the reversal potential towards more negative values (Fig. 6E). This response is reminiscent of the NaI effect on the acid-induced Cl^−^ current (Fig. 4H, I), which was also not observed for the constitutive Cl^−^ current.

**Fig. 7:**
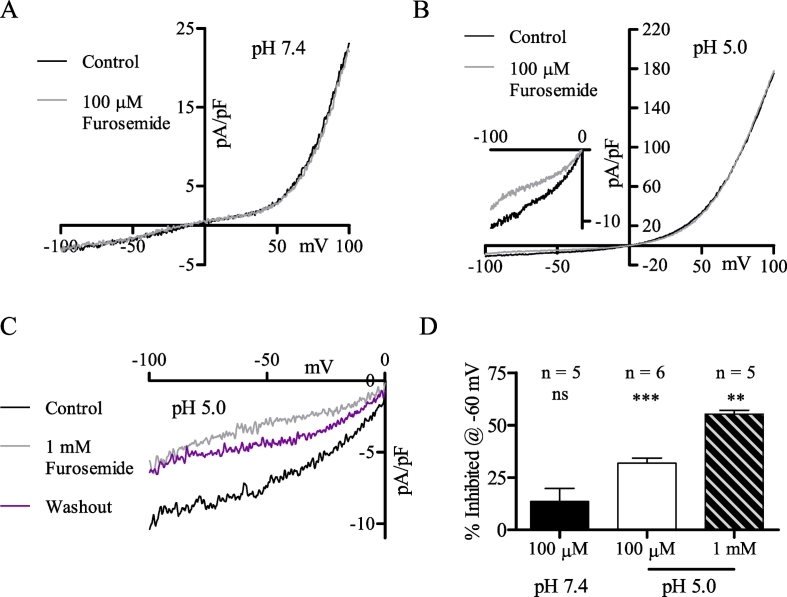
Furosemide modestly inhibits the acid-induced current. (A) Representative MDCT current before (black) and during (grey) maximal 100 µM furosemide blockade at pH 7.4 (oI). (B) Representative MDCT current before (black) and during (grey) maximal 100 µM furosemide blockade at pH 5.0 (oI). The inset is an expanded version of the I-V relationship from -100 mV to 0 mV. (C) Representative MDCT I-V relationship from -100 mV to 0 mV before (black) and during (grey) maximal 1 mM furosemide blockade at pH 5.0 (oI). Partial recovery from washout (violet) is also shown. (D) Mean percentage of MDCT current inhibited at -60 mV for the experiments shown in A (n = 5), B (n = 6), and C (n = 5). Currents were statistically compared via paired two-tailed Student's *t*-tests (mean ± SEM) and were considered significant when p < 0.05. ** refers to p = 0.01, *** to p = 0.001.

The constitutive and acid-induced currents of MDCT cells were also distinguished by their responses to furosemide (Fig. 7), a loop-diuretic that inhibits Cl^−^ channels [34], [72]. Furosemide (100 μM) did not affect the constitutive current (Fig. 7A, D), but caused a modest yet statistically significantly reduction of the acid-induced current (Fig. 7B, D). This inhibition was greater at 1 mM (Fig. 7C, D). Somewhat surprisingly, statistically significant changes were only observed over the negative range of membrane potentials. Since positive potentials were unaffected and the changes at negative potentials were minor, it is difficult to conclude if furosemide affected the functionally dominant acid-induced Cl^−^ current.

### 3.7 The acid-induced Cl^−^ current is reduced by TRPM7 siRNA

**Fig. 8:**
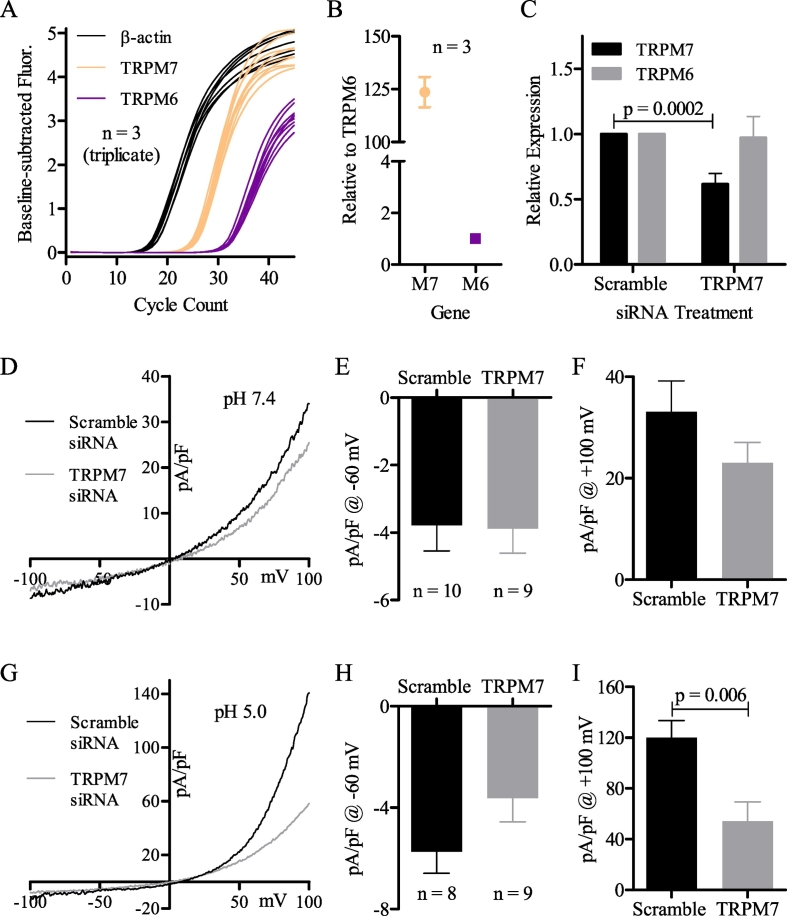
The acid-induced Cl^-^ current is significantly reduced by TRPM7 siRNA. (A) Amplification plot of ß-actin (black), TRPM7 (orange), and TRPM6 (violet) mRNA in MDCT cells (RT-qPCR; n = 3, triplicate). (B) Relative expression of TRPM7 to TRPM6 using the data shown in A. (C) Comparison of TRPM7 (black) and TRPM6 (grey) mRNA after 24 h transfection with scramble or TRPM7 siRNA, normalized to ß-actin (RT-qPCR; n = 4). (D) Representative MDCT current at pH 7.4 (oI) after transfection with scramble (black) or TRPM7 (grey) siRNA. (E) Mean current density of MDCT cells transfected with scramble (n = 10) or TRPM7 (n = 9) siRNA at -60 mV and pH 7.4. (F) Mean current density of MDCT cells transfected with scramble (n = 10) or TRPM7 (n = 9) siRNA at +100 mV and pH 7.4. (G) Representative MDCT current at pH 5.0 (oI) after transfection with scramble (black) or TRPM7 (grey) siRNA. (H) Mean current density of MDCT cells transfected with scramble (n = 8) or TRPM7 (n = 9) siRNA at -60 mV and pH 5.0. (I) Mean current density of MDCT cells transfected with scramble (n = 8) or TRPM7 (n = 9) siRNA at +100 mV and pH 5.0. Quantified mRNA was statistically compared by a Mann-Whitney *U* test (mean ± SEM). Currents were statistically compared via unpaired two-tailed Student's *t*-tests (mean ± SEM). Data were considered significant when p < 0.05.

The results indicate that MDCT currents are primarily permeable to anions, not cations. Consequently, it is perplexing that TRPM7 mRNA is expressed at a relatively high level in MDCT cells, 124 ± 7.1 fold that of DCT-localized TRPM6 [73] (Fig. 8A, B), yet macroscopic TRPM7 currents were not observed. One explanation is that TRPM7 may serve a regulatory role. Previously, it was suggested that the prominent effect of TRPM7 knockout was to reduce the expression of several other ion channels in mouse heart pacemaker cells [60], [61]. Using SMARTpool TRPM7 siRNA, we knocked down TRPM7 by 38.5 ± 8.2% after 24 h, as determined by RT-qPCR (Fig. 8*C*). For the MDCT currents, TRPM7 knockdown did not significantly affect the constitutive Cl^−^ current (Fig. 8D–F), whereas TRPM7 knockdown significantly reduced the acid-induced Cl^−^ current (Fig. 8G–I), but only over positive voltages (+100 mV; Fig. 8I).

### 3.8 Similar currents are present in mIMCD-3 cells

**Fig. 9:**
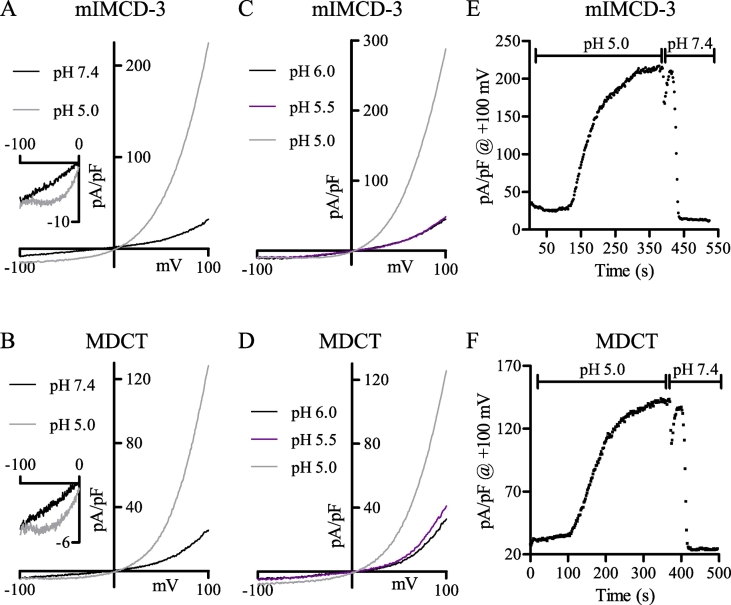
mIMCD-3 cells express similar macroscopic currents to MDCT cells. (A) Representative mIMCD-3 current at pH 7.4 (black; oI) and pH 5.0 (grey; oI). Expanded I-V relationship from -100 to 0 mV is presented in the inset. (B) Representative MDCT current at pH 7.4 (black; oI) and pH 5.0 (grey; oI). Expanded I-V relationship from -100 mV to 0 mV is presented in the inset. (C) Representative mIMCD-3 current at pH 6.0 (black; oI), 5.5 (violet; oI) and 5.0 (grey; oI). (D) Representative MDCT current at pH 6.0 (black; oI), 5.5 (violet; oI) and 5.0 (grey; oI). (E) Representative mIMCD-3 current during extracellular acidification (pH 7.4 to pH 5.0) and extracellular alkalization (pH 5.0 to pH 7.4) at +100 mV (oI). (F) Representative MDCT current during extracellular acidification (pH 7.4 to pH 5.0) and extracellular alkalization (pH 5.0 to pH 7.4) at +100 mV (oI).

Cells derived from the mouse inner medullary collecting duct (mIMCD-3), where the acidity of tubular filtrate can drop below the pH 5.5 required for activation of the acid-induced Cl^−^ current [3], [4], [5], were found to express similar currents to those found in MDCT cells (Fig. 9). This is particularly apparent for macroscopic currents recorded at pH 5.0 (Fig. 9A), which were essentially identical to those recorded in the MDCT cells (Fig. 9B). The acid-induced currents of MDCT and mIMCD-3 cells also had similar pH sensitivities, as both only activated at pH < 5.5 (Fig. 9C, D). Moreover, mIMCD-3 and MDCT currents showed similar temporal responses when extracellular pH was shifted from pH 7.4 to pH 5.0 (slow, ~ 250 s), and returned back to pH 7.4 (fast, ~ 20 s) (Fig. 9E, F).

## 4 Discussion

It was previously reported that NCC blockade by thiazides hyperpolarized MDCT cells in an NPPB-sensitive manner, implicating NPPB-sensitive Cl^−^ channels [21]. In the present study, we describe two dominant NPPB-sensitive outwardly rectifying Cl^−^ currents in MDCT cells: the constitutive Cl^−^ current and the acid-induced Cl^−^ current. We further show similar outwardly rectifying and acid-induced currents in mIMCD-3 cells, which correspond to a region of the renal tubule where the pH is capable of activating the acid-induced Cl^−^ current.

Due to the reported difficulty of microdissecting the mouse DCT [20], [37], MDCT cells are regularly used in studies examining the NCC, as they express the thiazide-sensitive NCC and various interacting proteins [2], [20], [21], [23], [37]. The recent genesis of an MDCT subclone that highly expresses the NCC further emphasizes the applicability of these cells to NCC related studies [9], [36], [37], [58].

Regarding the MDCT Cl^−^ currents, our results suggest that they do not arise from ClC-K2/barttin. Biophysically, ClC-K2/barttin is inhibited by extracellular acidification, activated by [Ca^2+^]_o_ (maximally at > 100 mM), and has a selectivity sequence of Cl^−^>Br^−^>I^−^ [16], [46], [66]. This contrasts the constitutive Cl^−^ current, which is inhibited by 110 mM [Ca^2+^]_o_ and has a selectivity sequence of I^−^>Br^−^≥Cl^−^. This also contrasts the acid-induced Cl^−^ current, which is potentiated by extracellular acidification and has a selectivity sequence of I^−^>Br^−^>Cl^−^.

**Fig. 10:**
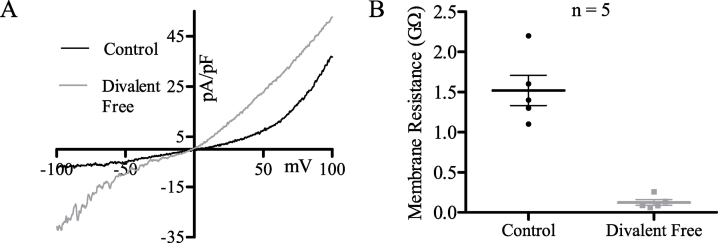
MDCT currents are divalent inhibited at pH 7.4. (A) Representative MDCT current with (black; oI) and without (grey; oXIV) extracellular divalent cations. (B) Plot of total membrane resistance (GO) in control (black) and divalent cation free (grey) extracellular solutions (n = 5). Total membrane resistance is presented as mean ± SEM.

An unusual characteristic of the MDCT constitutive Cl^−^ current is its prominent inhibition by > 100 mM [Ca^2+^]_o_. While Ca^2+^ inhibited Cl^−^ currents are rarely reported, one has been described in Xenopus oocytes, and it was observed that current magnitude increased in divalent cation free extracellular solutions [76]; a response not unique to Cl^−^ currents [18], [30], [50], [70]. We note a similar result for MDCT cells recorded in a divalent cation free extracellular solution (oXIV) (Fig. 10*A*). However, as this increase was coupled to a dramatic decrease in total membrane resistance (Fig. 10*B*), it is difficult to conclude if this response is due to enhanced activation of the constitutive Cl^−^ current.

Acid-induced Cl^−^ currents have been reported in many cells [1], [8], [19], [40], [41], [47], [48], [53], [64], [65], [75], [78], although only those from cortical neurons, HeLa cells, human embryonic kidney (HEK) 293 cells, and human bronchial epithelial (HBE) cells share the anion selectivity sequence we report for the MDCT acid-induced Cl^−^ current (I^−^ > Br^−^ > Cl^−^) [8], [41], [53], [65], [75]. However, the acid-induced Cl^−^ current of MDCT cells has two distinguishing characteristics. First, it is time-independent and fast activating, whereas the acid-induced anion currents of HeLa, HEK293, and HBE cells are time-dependent, activating slowly in response to voltage steps [8], [41], [75]. Second, the MDCT current requires nearly 250 s of exposure to a pH 5.0 extracellular solution to reach peak current magnitude, whereas the acid-induced anion currents of cortical neurons, HEK293, and HBE cells reach peak levels as quickly as 20–30 s [8], [41], [53], [65]. This slower growth of current in MDCT cells cannot be attributed to slow perfusion, as reintroduction of a pH 7.4 extracellular solution reduced MDCT currents in < 20 s. These results indicate that the acid-induced Cl^−^ current of MDCT cells is distinct from previously reported acid-induced anion currents.

The highly acidic activation threshold of the acid-induced Cl^−^ currents is unlikely to be realized under physiological conditions in most tissues. As a result, theories evaluating the potential roles of these currents have focussed on pathophysiology, including cell swelling and acidosis-induced necrotic cell death due to Cl^−^ influx [65], [75]. In the present study, we observed that the acid-induced Cl^−^ current requires an extracellular pH < 5.5 to activate, which can occur in the IMCD [3], [4], [5]. Thus, an important conclusion of this study is that mIMCD-3 cells express an acid-induced current with similar biophysical properties to the acid-induced Cl^−^ current found in MDCT cells. In this region, a Cl^−^ current is likely to secrete [32], and therefore necrotic cell death due to Cl^−^ influx is unlikely. In addition, the discovery that pH sensitivity is right-shifted (to less acidic values) at 37 °C [64] increases the likelihood that acid-induced Cl^−^ currents would have a physiological role in the distal tubule.

An intriguing observation of this study is that TRPM7 knockdown significantly reduces the acid-induced Cl^−^ current, but does not significantly affect the constitutive Cl^−^ current. This could be attributed to a regulatory role of TRPM7, as TRPM7 knockdown was shown to alter the mRNA abundance of several other ion channels in heart cells [60], [61], or could be attributed to cell stress, as TRPM7 knockdown is known to reduce cellular viability [33], [67]. Whatever the mechanism, the conclusions derived from the siRNA experiments support the notion that the acid-induced Cl^−^ current and the constitutive Cl^−^ current arise from different ion channels, since TRPM7 mediated regulation or cell stress should have affected both currents equally if they arose from one channel.

In conclusion, our results demonstrate that MDCT cells express two dominant NPPB-sensitive Cl^−^ currents, which due to their unique biophysical and regulatory properties are considered novel. We hypothesize that these Cl^−^ currents may participate in a Cl^−^ feedback cycle since intracellular Cl^−^ depletion is an activator of the NCC [55], and blockade of the NCC hyperpolarizes the plasma membrane in a response that is dependent on NPPB-sensitive Cl^−^ channels [21].

## Funding sources

This study was funded by grants from the Canadian Institute of Health Research (CIHR-MOP-57786 and CIHR-MOP-133451). RMT was supported through a Canada Research Chair/Canadian Foundation for Innovation award and British Heart Foundation Chair (29762).

## Transparency document

Transparency document. Image 1
